# When AI feels more human: language and user experiences with ChatGPT

**DOI:** 10.1186/s40359-026-04934-3

**Published:** 2026-06-15

**Authors:** Merve Armagan-Bogatekin

**Affiliations:** 1 Sağlık Bilimleri University, İstanbul, Turkey; 2 İbn Haldun University, İstanbul, Turkey

**Keywords:** Media Equation Theory, Human–AI Interactions, Anthropomorphism, ChatGPT, Multilingual Communication, inclusivity

## Abstract

**Purpose:**

This study examines how using a native versus a foreign language shapes users’ perceptions of ChatGPT in terms of linguistic proficiency, cultural fluency, emotional connection, and perceived human likeness. Drawing on Media Equation Theory, the study explores how multilingual contexts influence human–AI interaction and users’ interpretations of humanlike qualities in AI communication.

**Design/methodology/approach:**

Participants interacted with ChatGPT in both their native language (Turkish) and a foreign language (English). Following these interactions, semi-structured interviews lasting approximately 30–45 min were conducted, audio-recorded with participants’ consent, and transcribed verbatim. The data were analyzed using Interpretative Phenomenological Analysis (IPA). Through an iterative coding process, three main themes and nine sub-themes were identified.

**Findings:**

Language plays a significant role in shaping perceptions of AI interaction. Participants with lower English proficiency attributed greater humanlike qualities to ChatGPT and reported a stronger emotional connection when interacting in Turkish. However, interactions in English were generally perceived as more efficient and intelligent. Participants also raised concerns regarding ChatGPT’s cultural fluency, particularly when culturally specific expressions were not adequately reflected. Overall, the results suggest that multilingual users may experience a tension between emotional connection and perceived AI competence across languages.

**Research limitations/implications:**

The study involved 33 bilingual participants, and the findings may not be generalizable beyond this particular context. Nevertheless, the results highlight the importance of linguistic and cultural context in shaping human–AI interaction and suggest that multilingual environments may influence how users evaluate AI systems.

**Practical implications:**

Findings suggested a linguistic-emotional trade-off among non-native English-speaking users: participants commonly described Turkish-language interactions as fostering greater emotional connection and relational closeness, whereas English-language interactions were more often perceived as more competent and intelligent. The findings indicate that improving linguistic accuracy and cultural responsiveness in AI systems may enhance inclusivity, perceived empathy, and user experience in multilingual contexts.

**Originality/value:**

This study contributes to the growing literature on human–AI interaction by examining how multilingual users perceive AI systems across languages and by demonstrating how linguistic background shapes emotional and social perceptions of AI communication.

ChatGPT, a large language model developed by OpenAI and released in 2022, has rapidly gained global attention for its ability to generate human-like conversational responses. ChatGPT’s ability to generate humanlike responses and meaningful dialogues as a conversational system was one of the most important reasons for this attention. Therefore, interactions between AI tools and humans attracted directed research focus to explore the psychological processes underlying human–AI interaction [1]. However, there is a gap in the literature on the use of these technologies in bilingual or multilingual contexts in Türkiye, despite the fact that linguistic background can profoundly influence people’s experiences and perceptions. Research shows that language-specific factors can impact how people relate to AI [2]. Thus, investigating this phenomenon across different languages could help develop more inclusive AI systems.

The current study is based on Media Equation Theory (MET), which investigates how people usually treat and perceive media and technological tools. The theory suggests that people are likely to treat these tools as social actors and attribute humanlike qualities to them [3]. Using this theory, the goal was to explore how linguistic and cultural contexts can change people’s perceptions and responses to artificial intelligence.

## Literature review

ChatGPT is a large language model-based conversational agent developed by OpenAI, capable of generating contextually coherent, human-like responses across a wide range of communicative tasks. AI systems are designed to process information, recognize patterns, and generate outputs that simulate aspects of human communication and reasoning. Unlike human cognition, however, these capabilities are grounded in statistical modeling of large datasets rather than genuine understanding or learning [4].

Even though AI has many benefits, it raises ethical concerns and risks, including the potential to prolong existing inequalities. AI might perpetuate inequalities through biases and discriminatory decision-making processes [5]. Therefore, interactions with tools like ChatGPT have become an essential research topic in social psychology since this field focuses on social situations and interactions [6]. As a result, scholars have increasingly turned their attention to understanding relational and attachment processes in human–AI and human–robot interactions.

Although research on attachment and relational processes has been predominantly developed in the context of Human-Robot Interaction (HRI), the theoretical frameworks emerging from this body of work are increasingly applicable to conversational AI systems such as ChatGPT. Both HRI and human–AI interaction involve sustained, often anthropomorphized exchanges in which users may attribute social and emotional qualities to non-human agents. As conversational AI systems grow more sophisticated in simulating human-like communication (through natural language, responsiveness, and perceived empathy), the relational dynamics documented in HRI literature offer a relevant conceptual lens for understanding how users develop attachment and social bonds with systems like ChatGPT. It is therefore warranted to draw upon HRI-informed frameworks when examining relational processes in conversational AI contexts.

Following this line of knowledge on HRI in the field, Mitchell and Jeon [7] conducted a systematic review by using the PRISMA framework. They included 30 studies published in the last 15 years and focused on attachment within Human-Robot Interactions (HRI). Their results showed that prolonged interaction time, giving robots human-like names, and communication skills, such as speech, gestures, and emotional expressions, were important for fostering emotional attachment. While the first two factors can be established relatively easily, communication-based interaction requires linguistic fluency. This requirement may disadvantage non-native English speakers, as many AI systems are primarily trained on English-language datasets. According to Hanna and colleagues [8], biases in AI systems can stem from issues such as training data, algorithmic bias, feature engineering, and selection issues.

While certain languages, such as Turkish, are not categorized as underresourced, only a few research groups focus on them [9, 10]. Many Turkish datasets rely on machine translation from English sources, which can introduce artifacts and quality concerns. Existing Turkish benchmarks often rely on translations from English sources, which fail to capture the linguistic and cultural nuances of Turkish, leading to inconsistent performance [11]. Domain-specific Turkish AI applications also show progress and persistent gaps. In the biomedical domain, BioBERTurk represents an effort to develop Turkish biomedical language models, but researchers note the limited availability of Turkish medical texts in a low-resource setting [12, 13]. Studies also showed that educational applications initially struggled to generate coherent and contextually appropriate Turkish questions [14].

Turkish speech recognition has advanced with datasets like the Turkish Speech Corpus, but most automatic speech recognition efforts have focused on higher-resourced languages like English, Mandarin, and Japanese [15]. Neural machine translation models for the English-Turkish language pair face challenges due to the morphological richness and structural divergence of the Turkish language [16]. In cross-lingual relation classification, Turkish performance lags behind high-resource languages. The RELX dataset, which covers English, French, German, Spanish, and Turkish, enables systematic cross-linguistic comparison; however, Turkish consistently shows lower performance [17].

Large language model performance on Turkish tasks consistently lags behind English benchmarks. While these scores demonstrate substantial capabilities, they fall short of human-level comprehension and lag behind the performance these same models achieve on English benchmarks [17]. Turkish consistently underperforms English [9, 16–18].

These gaps persist despite recent advances in Turkish language modeling and cannot be attributed solely to evaluation methodology or task design. They primarily reflect genuine limitations in current Turkish AI capabilities, with real-world consequences for Turkish speakers’ access to AI-powered services and applications. These structural limitations in Turkish language AI capabilities are not merely technical shortcomings; they carry direct social-psychological consequences for many non-native English speakers who rely on AI systems for both everyday and expertise-dependent tasks. Understanding how such disparities shape users’ perceptions, emotional responses, and sense of connection to AI systems, therefore, requires a social-psychological framework. There is a significant body of literature on the social-psychological perspective of human-AI interactions, beginning with Weizenbaum’s observations about the social effects of his chatbot, ELIZA [19, 20]. MET, on which the current study is based, is another important milestone in the field.

### Media equation theory

MET proposes that people tend to treat media outlets such as television and computers as though they were human beings [3]. According to the theory, humans apply the same social norms and behaviors to their interactions with technology as they do with other individuals. People unconsciously consider computers genuine interactants and extend rules of politeness and bias to those machines [3]. Gambino and colleagues [21] also discussed the reverse effect. They argued that interactions with these tools could also shape human interactions and affect how individuals treat others. Therefore, interacting with technology and media tools significantly influences us as human beings.

Reeves and Nass [3] advocated that people attribute personality traits, gender, and social roles to machines. People are also more likely to attribute humanlike qualities (i.e., emotions, intentions, cues) to non-human entities. Thus, when chatting with an AI system like ChatGPT, people may interact with it as if it were a human and exhibit social behaviors such as politeness, empathy, and reciprocity. They might also apply social rules, such as courtesy.

Empirical research has since extended MET beyond traditional media contexts into conversational AI and chatbot interactions. Araujo [22] demonstrated that chatbots employing human-like language cues (such as a human name and a natural conversational tone) elicited significantly greater anthropomorphism and emotional engagement among users, providing empirical support for MET’s applicability to text-based AI systems. Similarly, Nass and Moon [23] showed that users applied gender stereotypes and social expectations to purely text-based computer agents, suggesting that the social responses predicted by MET do not require visual or embodied cues and are therefore relevant to language-driven interactions with systems like ChatGPT.

However, it is worth noting that MET was originally developed in the context of passive media consumption, and its application to generative AI introduces new theoretical considerations. Unlike television or static computer interfaces, conversational AI systems such as ChatGPT engage users dynamically through language, making linguistic quality and cultural alignment central to users’ perception of the system as humanlike. This distinction is particularly consequential for non-native speakers, for whom the AI’s linguistic and cultural performance in their native language may serve as the primary cue for social and emotional engagement.

This framework is helpful for social psychologists to explore the social dynamics of human-AI interactions. We use MET to investigate bilingual perceptions of these interactions, as our expectations and emotional responses may vary across cultures and languages. By applying MET, we aim to examine how users’ social norms differ when engaging with the system in their native language versus a foreign language.

According to this theory, using their native language would make participants treat the AI as more natural, familiar, and empathetic—in other words, more ‘humanlike’. They would expect the AI to reflect the deep cultural nuances of their native language. On the other hand, if participants interact in a foreign language, they are expected to have different expectations and reactions, leading to less emotional engagement and a less human-like perception. Therefore, it can be inferred that AI tools are becoming social actors in today’s world.

### AI: a social actor

Linnemann and Reimann [6] suggest that AI can be perceived as a social actor, and individuals imagine or experience the presence of other people during their interaction with AI. They also argued that Social Learning Theory can be applied to AI systems, as it holds that people observe and imitate others to learn. AI-powered systems do the same thing, since they can model and reinforce certain behaviors. Furthermore, AI systems are individualized and flexible, and they do not expect reciprocity. Therefore, they can diminish social skills since no other human being can provide such a nurturing environment for others. This is an ethical issue to consider.

Another ethical consideration is that AI mechanisms often inherit underlying biases from historical discrimination and from flawed algorithms and datasets used to train them [4]. It is recommended that diversity and inclusion principles be embedded in the development of AI technologies to address concerns about trust, transparency, and algorithmic oppression [5, 24].

ChatGPT is more exciting than other AI tools because humans train it and reinforce its learning [25]. It also allows interaction in multiple languages, which might help undermine the dominance and privilege of native English speakers in many domains [26]. AI tools can help diminish long-standing inequalities when appropriately trained, but can also perpetuate them if they are not culturally adaptable. Ghio [26] also argues that perpetuating an English-dominant culture is evident even at the design stage, as the number of people providing feedback to ChatGPT in languages other than English is limited. Therefore, in this study, we sought to document whether ChatGPT was perceived as performing better in English by Turkish-speaking users.

### Inclusive and responsible AI

English is already the dominant language in publications, conferences, and reviews in the field of psychology, creating cultural and linguistic barriers for non-English-speaking communities [27, 28]. This linguistic dominance extends beyond academic discourse to AI systems themselves, where the underrepresentation of non-English languages in training data risks reproducing and amplifying existing inequalities. AI tools can help diminish long-standing inequalities when appropriately trained, but can also perpetuate them if they are not culturally adaptable. Therefore, it is crucial to focus on making AI more inclusive and on addressing the needs of marginalized groups, rather than exacerbating systemic inequalities.

There is a gap in the literature on this issue for Turkish-speaking users, as this technology is relatively new. Lai and colleagues [2] reported that ChatGPT performs better with English prompts than with prompts in other languages. This was especially true for complex reasoning skills, including summarizing information. They conducted the study in 37 different languages, including Turkish. Lai et al. [2] used the ratio of data for each of these 37 languages in the CommonCrawl corpus, which is the primary dataset used to pre-train GPT-3. Then, they classified the resource levels for each language. They considered languages as high-, medium-, low-, and extremely low-resource based on the data ratio (i.e., greater than 1% (> 1%), between 0.1% and 1% (> 0.1%), between 0.01% and 0.1% (> 0.01%), and smaller than 0.01% (< 0.01%) respectively). They classified Turkish as a medium-resource language in the study.

Their results showed that ChatGPT performed reasonably well in Part-of-Speech (POS) Tagging for Turkish, a foundational task that underpins grammatical accuracy in generated text. However, it struggles with Named Entity Recognition (NER), making it difficult to identify entities such as person names, locations, and organizations in Turkish text. From a user experience perspective, this results in responses that may feel imprecise or contextually disconnected.

Their results also showed poor performance in Turkish Natural Language Inference (NLI). NLI is the ability to determine logical relationships between sentences, and it might affect the coherence and reliability of responses users receive, potentially undermining perceptions of the system’s intelligence. ChatGPT in Turkish also performed poorly on Question Answering (QA), suggesting that users seeking accurate, contextually grounded answers in Turkish are likely to encounter responses that feel less competent and less trustworthy than those generated in English.

They also found that ChatGPT’s performance on Common Sense Reasoning (CSR) tasks in Turkish is relatively low (49.4%), which is particularly consequential for naturalistic conversation. When an AI fails to apply everyday cultural and contextual knowledge, users may perceive it as less humanlike and more machine-like.

Taken together, these findings suggest that Turkish-speaking users are likely to experience ChatGPT as less intelligent, less reliable, and less humanlike in their native language. It happens not as a reflection of their own linguistic competence, but as a structural consequence of unequal resource allocation in AI development.

Overall, ChatGPT in Turkish performs moderately across most tasks, but its performance is capped compared to high-resource languages or fine-tuned supervised models. When a language model performs less accurately, produces culturally misaligned outputs, or fails to capture the affective nuances of a user’s native language, it may alter the nature of the human–AI relationship.

Therefore, we aimed to deepen these quantitative findings through a qualitative exploration of the issue, focusing on the experiences of Turkish-English bilingual speakers. In this regard, MET helps explore lower social expectations and higher cognitive distance while engaging with ChatGPT in a foreign language that people feel less comfortable with. This will explain how language proficiency and cultural fluency influence AI’s perceived humanlike or machine-like qualities. This understanding will help emphasize the need to create authentic, inclusive, and engaging AI systems sensitive to all needs.

Our aim is to understand how bilingual users perceive ChatGPT’s human likeness in English and Turkish, what social and emotional responses emerge when using ChatGPT in both languages, and how cultural fluency affects trust and engagement with ChatGPT across languages. Drawing on MET, several conceptual propositions guide the present inquiry. First, if users unconsciously apply social norms and emotional scripts to AI systems as they do to human interlocutors, then the language of interaction may modulate the depth of emotional engagement, with native language interactions potentially activating stronger affective responses and a greater sense of connection (RQ1).

Second, MET’s premise that humanlike cues trigger social perception suggests that AI’s linguistic fluency and conversational naturalness may shape how users perceive and interpret humanlike qualities during interactions with ChatGPT (RQ2).

Third, given that MET posits the extension of social expectations (including competence and credibility judgments) to media and technology, users may evaluate ChatGPT’s cultural fluency as a marker of trustworthiness and intelligence. Thus, there might be misalignments between the AI’s outputs and users’ cultural norms, potentially undermining both trust and perceived intelligence (RQ3).

These propositions do not constitute testable hypotheses in the conventional sense; rather, they provide a theoretically informed lens through which participants’ lived experiences of bilingual AI interaction are examined. Therefore, our research questions are as follows:

- RQ1: How does the language used in interactions with ChatGPT (native vs. foreign language) influence users' social and emotional responses, as conceptualized within the Media Equation Theory framework?
- RQ2: How do users describe and interpret humanlike qualities in their interactions with ChatGPT?
- RQ3: How does ChatGPT's cultural fluency in the language of interaction influence users' social expectations, trust, and perceived intelligence of the system?

## Methodology

### Participants

This study’s participants were adults who were Turkish speakers of English (as a foreign language). Another inclusion criterion was regularly interacting with AI systems, such as virtual assistants or chatbots. We used both passive/indirect methods, such as flyers, and active/direct strategies, including person-to-person interactions, to recruit participants. Recruitment followed a snowball sampling approach, in which initial participants referred eligible individuals through their personal and professional networks. Thus, we used a multimodal recruitment approach, combining active and passive strategies, as suggested by Negrin and colleagues [29]. All interviews were conducted face-to-face in a quiet, private setting to ensure participant comfort and data quality.

There were 33 participants aged 19 to 43. Data collection continued until interpretive sufficiency was achieved. Although IPA conventionally employs smaller samples to enable deep idiographic analysis, a growing body of IPA scholarship has demonstrated the methodological viability of larger samples, particularly when the research aims to capture experiential diversity across a defined group [30]. The present study’s sample of 33 participants reflects the dual requirements of its hybrid design, which incorporates both qualitative (IPA) and quantitative (correlational) components.

There were 15 female (45.5%) and 18 male (54.5%) participants. All participants were proficient in English, as required by the language-based task. This was a prerequisite mentioned in the flyers. The self-perceived English proficiency ranged from upper-intermediate to native-like levels. Another recruitment criterion was familiarity with generative AI tools. That is why one participant was excluded from the study, as they lacked prior experience with AI tools. The Institutional Review Board approved the study at Ibn Haldun University (Approval No. 2024/09 − 06).

### Data collection

Data collection followed a structured two-phase protocol. In the first phase, participants interacted with ChatGPT in both Turkish and English using standardized prompts. All sessions were conducted face-to-face in a quiet, private setting. Participants first interacted with ChatGPT in Turkish for approximately five minutes using the following prompt:

1. Prompt for a chat in Turkish: “I have a big deadline coming up, and I am feeling really overwhelmed. How can I manage this stress?” (In Turkish: “Önemli bir son teslim tarihim var. Ve gerçekten bunalmış hissediyorum. Bu stresi nasıl yönetebilirim?“).

Following a brief pause, participants then interacted with ChatGPT in English for approximately five minutes using the following prompt:

2. Prompt for a chat in English: “I am struggling to stay motivated at work/school. Do you have any tips to help me focus?” (In Turkish: “İşte/okulda motive kalmakta zorlanıyorum. Odaklanmama yardımcı olacak öneriler var mı?“).

In the second phase, trained research assistants conducted semi-structured interviews with participants immediately following the ChatGPT interactions. The interviews were based on participants’ experiences during both language interactions and lasted approximately 30–45 min. All interviews were audio-recorded with participants’ consent and transcribed verbatim. All participants signed an informed consent form prior to the start of the session. The total participation time, including both the ChatGPT interaction and the interview, was approximately 40–50 min.

### Data coding and analysis

After data collection, interpretative phenomenological analysis was used to analyze the dataset. The qualitative data were analyzed using the principles of Interpretative Phenomenological Analysis (IPA), which focuses on participants’ lived experiences and the meanings they attribute to them. For the correlational analysis, SPSS 25 software was used. The present study employs a hybrid design that incorporates both qualitative and quantitative components. Each component is evaluated against the methodological standards specific to its own epistemological paradigm, consistent with established practice in mixed-methods research [31]. This dual-paradigm approach reflects the study’s aim to capture both the depth of individual lived experience and broader patterns across the sample.

The author read all interview transcripts several times to ensure familiarity with the content. During the initial reading, exploratory notes and preliminary codes were generated to capture experiential and semantic content directly from the data. After reviewing all transcripts, a codebook was prepared based on the identification of common themes. It was an iterative process, as we revisited participants’ responses to ensure accuracy and consistency. The codebook was generated using an inductive approach, meaning codes emerged from participants’ accounts rather than being imposed from a predetermined framework. Media Equation Theory informed the interpretation of the findings rather than the initial coding process.

Following the initial coding of a subset of transcripts, the preliminary codes were reviewed, compared, and refined through discussion during regular research group meetings. Codes that overlapped in meaning were consolidated, and those that did not meaningfully contribute to addressing the research questions were discarded.

The refined codes were then clustered into subordinate themes reflecting shared experiential content. These subordinate themes were subsequently reviewed across the full dataset and organized into superordinate themes representing broader patterns of meaning. This iterative process yielded a final thematic structure comprising three superordinate themes and nine subordinate themes. The revised codebook was then applied to the full dataset, and illustrative quotes were selected to represent each theme. Theme names were reviewed and refined for clarity and analytical depth.

Two Turkish research assistants who also speak fluent English (which helped with this study’s focus on local customs, practices, and cultural expectations) were trained for the coding procedure. This linguistic proficiency was significant for ensuring the coding process was accurate and that themes were not decontextualized. The shared linguistic and cultural background also helped establish a stronger rapport with participants, leading to more open and nuanced conversations during interviews.

Coding was conducted in multiple rounds. In the first round, the author and two trained research assistants independently coded a subset of transcripts to assess consistency and refine code definitions. During regular research group meetings, consistency among coders was assessed, and any discrepancies were discussed until a consensus was reached. The revised codebook was then applied to the full dataset. We also selected illustrative quotes and revised theme names for clarity and analytical depth.

As Brooks and Wearden [32] note, drawing on Yardley [33], inter-rater reliability measures in interpretive qualitative research merely yield an interpretation agreed upon by two people rather than serving as a genuine check on objectivity. This consensus-based procedure is consistent with established IPA practice, in which the aim of validity checks is not to prescribe a singular true account but to ensure the credibility of the final interpretation [32].

The phenomenological approach was selected specifically for this study because it focuses on lived experiences rather than on thoughts alone. As we asked participants to experience chatting in both languages for 10 min right before the interview, this method helped capture emotional nuances and cognitive responses. It also allowed us to explore in depth how their language shaped the interaction through semi-structured interviews. Participants were encouraged to reflect on their thoughts and feelings to explore their emotional engagement with ChatGPT. This method does not rely solely on surface-level answers; deep reflections are appreciated.

A correlation analysis was performed to examine potential relationships between quantified qualitative themes and demographic variables. Qualitative themes were converted into numerical variables by counting the frequency of each coded theme within participants’ responses. The analysis was based on the full sample (*N* = 33). Given the exploratory nature of the analysis, no correction for multiple comparisons was applied. The correlations were conducted as exploratory analyses to identify potential associations between themes and demographic variables.

## Results

### Themes

Three main themes and nine sub-themes emerged after reviewing the transcripts (see Table 1).

**Table 1:** Themes and explanations for emotional and linguistic attachment in ChatGPT interactions

Main Theme	Sub-Theme	Explanation	Example Quote
Emotional Connection	In Turkish	The participant feels more emotionally connected when communicating in Turkish.	“I felt more sincere in Turkish.”
	In English	The participant feels more emotionally connected when communicating in English.	“I felt more sincere in English.”
	General Emotional Connection	Participants describe emotional responses (such as anger, happiness, or excitement) that emerged during ChatGPT interactions, regardless of language, reflecting the affective dimension of human–AI engagement more broadly.	“When it feels like just a page from a book, it loses its sincerity.”
Perceived Intelligence	In Turkish	The participant perceives ChatGPT as more efficient and intelligent when using Turkish.	“I use it for homework, research, etc., in Turkish.”
	In English	The participant perceives ChatGPT as more efficient and intelligent when using English.	“I use it for homework, research, etc., in English.”
	Linguistic Fluency	Comments on grammar, fluency, or linguistic accuracy influence perceptions of ChatGPT’s intelligence.	“The suggestion it gave always felt like something I’d gotten from a website.”
Perceived Human-Likeness	Anthropomorphic Attribution	Participants describe ChatGPT as resembling a human (psychologist, professor, friend).	“It feels like a psychologist.”
	Uncanny Valley	Participants feel uneasy about ChatGPT’s almost-human yet not fully natural responses.	“I can still feel the coldness of those words.”
	Social and Cultural Norm Adherence	Participants treat ChatGPT as they would a human, following politeness and cultural norms.	“It is based on Western sources (…). I need to provide some context and some information so that AI can understand and address.”

Below, you will find a frequency table for each theme (See Table 2).

**Table 2:** Frequencies of Sub-themes identified in the study

Theme	Frequency
Emotional Connection in Turkish	119
Emotional Connection in English	29
Perceived Intelligence in Turkish	46
Perceived Intelligence in English	159
Social Norm Adherence	58
General Emotional Connection	58
Linguistic Quality and Grammar	54
Uncanny Valley	41
Anthropomorphic Attitudes	125

### Perceived humanlikeness

The first main theme relates to ChatGPT’s comparison to humanlike figures. There were three sub-themes under this theme: Anthropomorphic Attribution, Uncanny Valley, and Social Norm Adherence. The operational definition for Anthropomorphic Attribution is AI’s ability to generate human-like text, engage in coherent dialogues, and answer complex questions that are indistinguishable from those of humans [34, 35]. In the study, 125 instances were coded as HUM. This includes mimicking conversational patterns, emotional responses, and professional roles.

Turkish participants in this study frequently attributed human qualities to ChatGPT and even perceived ChatGPT not just as a tool but as a conversational partner. Several participants wished ChatGPT could establish a humanlike connection with them. These instances were coded as anthropomorphism. A study related to this finding used ChatGPT and trust games, and the results showed that participants had higher trust in humanoid AI conditions than in non-humanoid AI conditions [35]. However, the current study expands this by illustrating how language choice mediates the strength of anthropomorphization. Many participants in our study described their interactions with ChatGPT as resembling those with a person, such as a psychologist, professor, or friend. This indicates how much users anthropomorphize the AI in their minds. For example, one participant said:

*“I tell ChatGPT: Speak to me more like a human*,* more human-ish*,* talk to me more humanely*,* like a psychotherapist*,* do not use headings*,* don’t list things*,* do not bullet point*,* just talk like this.”*

The participant explicitly requested the AI to avoid mechanical formats such as bullet points or headings, reflecting a preference for emotionally engaging, conversational language over information delivery alone. When asked, “If ChatGPT were a person, what would it be like?“, one participant responded:

*“What they are trying to present to us is this very sweet*,* almost humble*,* helpful AI*,* but for some reason*,* every time I talk to it*,* I get a very arrogant vibe. I mean*,* it gives information*,* the information is with it*,* and you are trying to learn how to ask for that information from it. Also*,* when I speak in Turkish*,* I find it ignorant. Of course*,* when I assign these labels to it*,* I also need to consider the different things in my own subconscious*,* like how I see myself or how I perceive Turkish people.”*

This response illustrates how users may attribute personality characteristics to AI systems, consistent with MET’s proposition that people apply social and emotional scripts to technological interlocutors. Notably, the participant also demonstrated reflexivity regarding their own interpretive process, acknowledging that their perceptions may be shaped by subconscious biases and cultural identity. The participant's perception that ChatGPT appears more competent in English but “ignorant” in Turkish reflects a broader hierarchy of knowledge production in which English is often positioned as the language of expertise, authority, and technological sophistication. Rather than merely evaluating the AI system's performance, the participant implicitly engages with historically embedded assumptions about linguistic prestige and legitimacy. This reflexive observation suggests that evaluations of AI are not solely responses to the technology itself but are also shaped by internalized cultural hierarchies and unequal global distributions of epistemic authority. This level of self-awareness is consistent with IPA’s emphasis on the co-constructed nature of meaning.

We also conducted a correlational analysis to determine whether the theme of human likeness correlates with other themes or demographic variables (see Table 3). To explore potential relationships between themes and demographic variables, qualitative codes were quantified by calculating the frequency of each theme within participants’ responses. These frequency counts were then used as variables in correlational analysis. This approach, sometimes referred to as quantitizing [36], is an established procedure in mixed-methods research whereby qualitative data are transformed into numerical representations to enable statistical analysis.

**Table 3:** Exploratory correlations between themes and demographic variables

Variables	*r*	*p*
Uncanny Valley – Emotional Attachment in English	0.47	0.006
Cultural Fluency – General Emotional Connection	− 0.41	0.019
Linguistic Fluency – Age	− 0.43	0.012
Emotional Connection – Self-reported English Level	0.47	0.007

The quantitizing process converts qualitative information into numerical values for statistical analysis [37]. The results showed a negative correlation between English proficiency and the perception of human likeness (*r = −* .55, *p* = .001).

### Uncanny valley

Another sub-theme that emerged here was “Uncanny Valley,” as many participants felt disturbed when ChatGPT’s responses were perceived as overly robotic, referring to interactions that felt mechanical, rigid, or lacking natural conversational flow. This feeling was specifically reported 41 times. Some participants liked AI’s responses to the questions but reported they were of insufficient quality, as with human interaction. One participant described:

*“Even though it tries to say things like ‘I” understand you*,* I’m trying to help you’ (laughs)*,* I can still feel the coldness of those words. because nothing can give you the warmth of looking into a person’s eyes*,* nothing can provide the warmth of personal interaction.“.*

This participant describes an experience that bears a structural resemblance to the Uncanny Valley effect [38], though in a linguistic rather than physical domain. The AI’s Turkish output was technically coherent, but its tone lacked the warmth and familiarity of genuine human interaction, leaving a sense of affective disconnection. While we do not claim this constitutes the classical Uncanny Valley phenomenon, the analogy captures the participant’s experience of responses that were near-human in form but not in feeling.

### Social norm adherence

The sub-theme of social norm adherence was selected since it was repeated 58 times by the participants. The operational definition of the term involves aligning one’s behavior with the expectations of one’s social environment [39]. Social norm adherence is relevant in human-AI interactions, too. Unfortunately, AI systems today lack moral agency and do not have the capacity for intentional norm adherence [40]. ChatGPT’s (in)ability to align with social expectations and cultural context in conversations was coded under this theme. Participants mentioned that the AI’s responses sometimes did not align well with their values, particularly when discussing culturally specific topics such as family, religion, tradition, or etiquette. For instance, in Turkish interactions, some participants expected ChatGPT to reflect values of respect and politeness in Turkish and Islamic culture:

*“It is like a foundational issue*,* because it is based on Western sources. It does not really align with my religion*,* Islam*,* from that perspective. I need to provide some context and some information so that it can understand and specifically address and focus on that point when responding. For example*,* we say ‘namaz’ (prayer)*,* but it suggests meditation instead. These kinds of differences*,* whether in English or Turkish*,* suggest practices like meditation and breathing exercises. At that point*,* it doesn’t really matter to me.”*

This quote shows how AI systems that ignore local religious expressions are perceived as detached or even irrelevant, even if the underlying intention remains the same. This quote suggests that users evaluate AI responses not only for functional accuracy but also for cultural and religious relevance. From the perspective of Media Equation Theory, such reactions indicate that users apply social and cultural expectations to AI systems, expecting them to demonstrate contextual awareness similar to that of a human interlocutor. When AI responses fail to acknowledge culturally meaningful practices, the interaction may be perceived as less authentic or less responsive to the user’s identity and worldview.

Another participant mentioned the difference between languages and the impact of word choices as follows:

*“In Turkish*,* there’s this difference—using ‘Tanrı’ or ‘Allah’ (…) Translating as ‘Allah’ made me feel that warmth in my language. However*,* if it were in English*,* it would most likely have translated as ‘God*,*’ and I would not have felt that warmth.”*

This quotation illustrates how subtle linguistic choices can shape users’ emotional responses to AI-generated content. Although the concepts of “God” and “Allah” may refer to the same theological entity, participants perceived them as carrying different emotional and cultural connotations. The use of “Allah” in Turkish evoked a sense of familiarity and warmth, whereas the equivalent English translation, “God,” felt more distant and less emotionally resonant.

This suggests that users evaluate AI responses not only for semantic accuracy but also for the cultural and emotional meanings embedded in language. From the perspective of Media Equation Theory, such reactions indicate that users apply socially and culturally grounded expectations to AI systems, expecting them to recognize contextually meaningful linguistic expressions. When AI responses fail to reflect these culturally embedded nuances, the interaction may feel less authentic or emotionally engaging.

### Emotional connection

Emotional connection was a central theme in this study, reflecting how participants felt connected to ChatGPT in English versus Turkish. There were three sub-themes under this main theme: Emotional Connection in English, Emotional Connection in Turkish, and Linguistic Fluency. Research identifies intimate behavior and self-disclosure as forms of emotional engagement, as they can evoke complex emotional responses among users. For example, personalization in AI interactions can enhance user experiences and foster deeper emotional connections [41]. The main theme was Emotional Connection, which covered emotional responses and general reactions without comparing languages. There were 58 instances of this theme coded in the study, and it was positively correlated with participants’ English proficiency (*r =* .47, *p* = .007).

There were two subthemes to differentiate between these linguistic contexts if the participant compares the two languages. This could explain the negative correlation between the emotion and culture themes mentioned in the previous section. Since sentences that did not mention a specific language and instead signified participants’ general emotional states toward ChatGPT were coded as EMO, these participants were less likely to provide culture-specific examples. For emotions in general (EMO), one participant said:

*“When it feels like just a page from a book*,* it loses its sincerity. Without that sincerity*,* it doesn’t really resonate. It’s not like having someone who can truly understand human emotions. If*,* through their writing*,* they could sense what emotional state they’re in at that moment and respond accordingly*,* I think that would make it much more useful.”*

This account points to a tension between informational adequacy and emotional responsiveness. The participant does not simply evaluate ChatGPT’s output as insufficient. Rather, they articulate a specific expectation: that the system should be capable of reading and responding to emotional states in real time. This expectation is itself significant, as it suggests the participant is applying the same relational standards to ChatGPT that they would to a human interlocutor. This pattern is consistent with MET’s proposition that users extend social scripts to technological agents [3]. The perceived absence of empathy thus functions not merely as a practical limitation but as a relational rupture, undermining the sense of connection the participant sought. A second participant elaborated on how this dynamic played out differently across languages:

*“In English*,* I asked it to talk to me like a friend; in Turkish*,* it only said ‘kanka’ (*MAB^1^: “bro” in Turkish) *because I asked for it. However*,* it started using street slang in English and called me ‘bro’. I wanted it to sound a bit warmer in both languages*,* but in Turkish*,* it did not quite do so. In English*,* instead of saying ‘I understand*,*’ it said ‘gotcha*,*’ and I think in that respect*,* the writing style was more sincere.”*

Here, the participant’s account draws attention to micro-linguistic cues (i.e., informal register, slang, and tone calibration) as carriers of emotional presence. What is analytically notable is that the participant made an equivalent request in both languages, yet experienced markedly different responses. This asymmetry suggests that ChatGPT’s capacity to modulate emotional tone is not uniform across languages, which aligns with Lai et al.‘s [2] finding that the system’s linguistic performance is structurally constrained in Turkish relative to English. From a MET perspective, the English system’s responsiveness to the participant’s social cues was sufficient to trigger a perception of greater warmth and sincerity, while the Turkish system’s failure to reciprocate disrupted this social dynamic. A third participant introduced a further layer of complexity, describing an ambivalence rooted in the cultural origins of the technology:

*“When I ask something in Turkish and get a response in Turkish*,* I like it*,* and it makes me feel more confident. However*,* the fact that the AI’s primary language is English and relies on many English-language sources — our preconception that it’s based on Western technology — gives me a sense of trust.”*

This account reveals a bifurcation in the participant’s emotional experience: native-language interactions generated a sense of comfort and confidence, while the perceived authority of English-based, Western-origin AI engendered a different kind of trust. The latter is rooted in competence rather than warmth. This distinction between affective trust and competence-based trust has implications for understanding how non-native English speakers navigate human–AI interaction, and resonates with the study’s broader argument that emotional connection and perceived intelligence may operate as partially independent dimensions of the user experience.

**Fig. 1:**
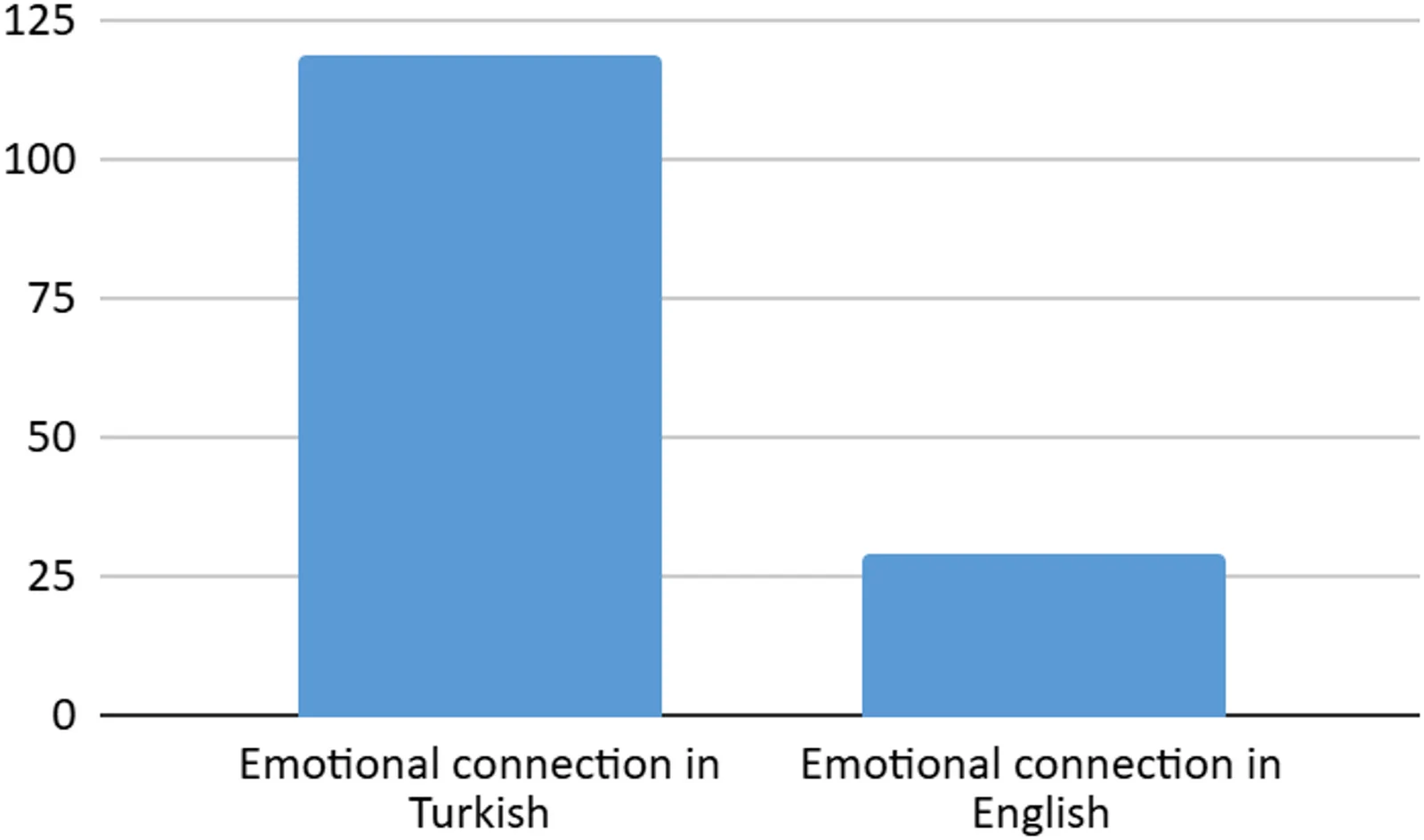
Emotional connection

Taken together, these accounts suggest that emotional connection in human–AI interaction is shaped not only by the language of interaction but also by the system’s capacity to respond to social and emotional cues within that language. Participants reported feeling more intimate and sincere when using ChatGPT in Turkish in 119 instances, suggesting that native-language interactions were more likely to activate the social and emotional responses predicted by MET. The score was only 29 for Emotional connection in English, the foreign language (see Fig. 1).

Furthermore, a positive correlation was found between emotional attachment in Turkish and perceived human-likeness, suggesting that these two dimensions of the user experience are mutually reinforcing. These findings suggest that when users interact in their native language, they are more emotionally open and relational, which in turn makes them more likely to view the AI as a humanlike entity. Taken together, these findings suggest that those with lower English proficiency prefer using ChatGPT in Turkish, which helps them build a strong emotional connection with the tool. Therefore, it might have helped those participants to perceive ChatGPT as more humanlike.

ChatGPT’s limitations in specialized fields that require expert knowledge might present a significant challenge for Turkish-speaking users. It can be challenging to achieve emotional satisfaction, establish trust, and access the necessary expertise in specialized subjects that demand in-depth knowledge and nuanced understanding for these populations. These limitations must be acknowledged, as they may limit disadvantaged users’ ability to benefit from such platforms.

### Perceived intelligence and efficiency

Another theme in this study was the perceived intelligence and efficiency in Turkish and English. Perceived intelligence in AI refers to the extent to which users attribute intelligent characteristics to AI systems. This perception can influence their interactions, acceptance levels, and behavioral intentions towards AI technologies [42].

**Fig. 2:**
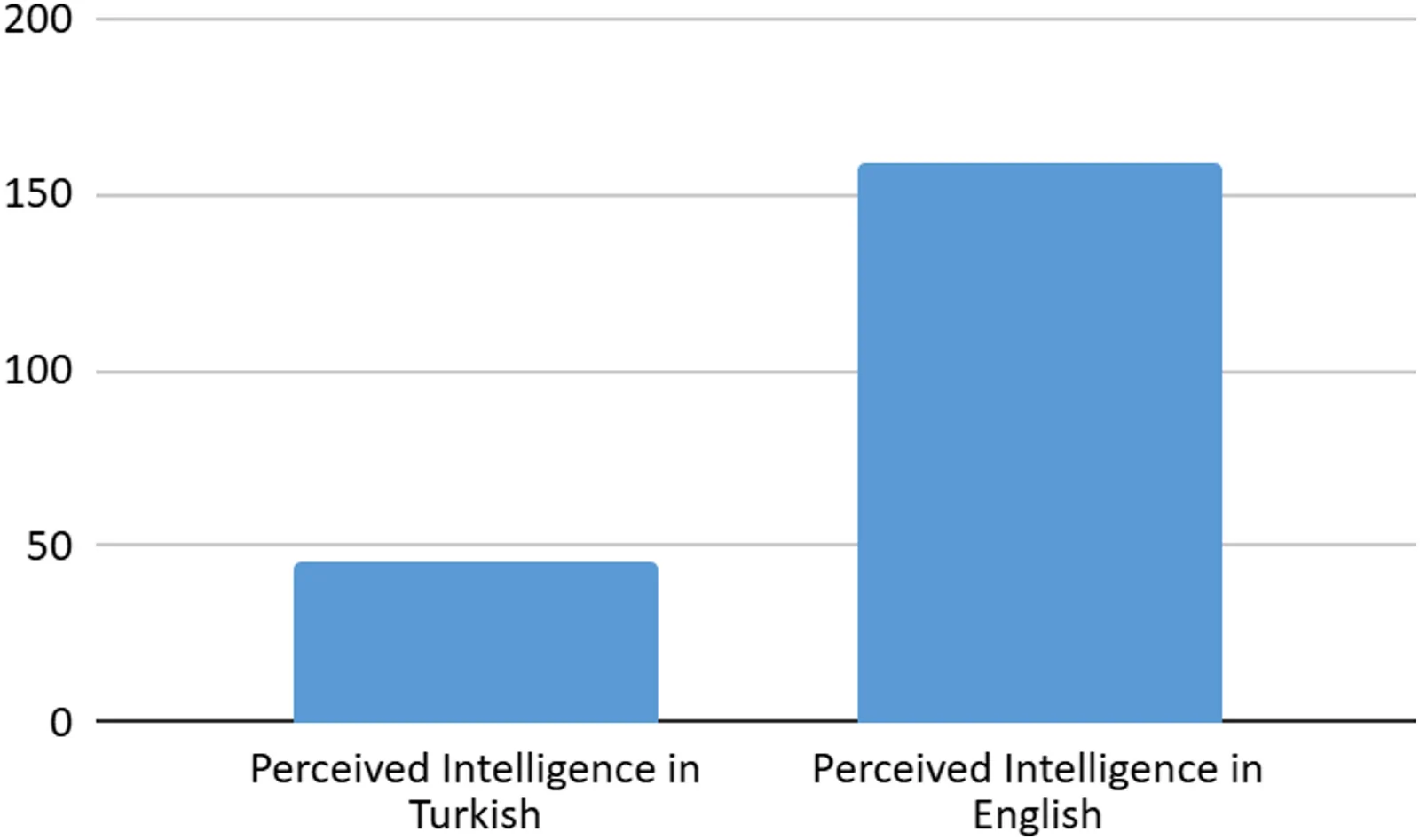
Perceived intelligence

Our results showed that ChatGPT was perceived as more intelligent and efficient when used in English, and there were 159 instances in which this was meant. The number was only 46 for Perceived Intelligence in Turkish (see Fig. 2).

Turkish performance in this study was acceptable but lacked depth, capability, and nuance compared to English performance. One participant used ChatGPT in Turkish for the first time during this study and stated that:

*“Because I started using it in English and always continued in English. I didn’t even think about using it in Turkish. I always thought its developers created it in English*,* so I assumed it didn’t even work in Turkish.”*

This quote shows an underlying technological bias among Turkish-speaking users. The participant mentions the belief that English is the “default” language of AI. This belief can lead to other languages being perceived as secondary or simply an extension of the system. Such assumptions can shape user expectations and trust even before an interaction begins.

Another participant said: *“When it makes mistakes in Turkish*,* I blame the AI (laughs)*,* but when it makes mistakes in English*,* I think*,* ‘I must have written it wrong.‘”* This asymmetry illustrates how language proficiency impacts perceived agency. Apparently, the user feels more in control and confident when interacting in their native language. Our results show that those who find Turkish more reliable or intelligent generally attribute this to limitations in their English proficiency, as they can read and understand Turkish responses with much greater accuracy and speed. For example:

*“I find it more reliable in my own language. Because when I write and read in Turkish*,* I read quickly. It often gives long responses*,* sometimes including every detail. So*,* I quickly skimmed it. In English*,* there are times when I might miss things*,* but in Turkish*,* for example*,* I can read almost everything—I would say with about 90%*,* maybe even 95% accuracy. That is why*,* in my opinion*,* Turkish works better.”*

This preference sounds less about ChatGPT’s linguistic quality and more about the cognitive ease and comprehension speed users experience in their native language.

### Linguistic quality and grammar

This sub-theme concerns how participants perceive ChatGPT’s interactions with itself. This sub-theme captures participants’ perceptions of the linguistic quality of ChatGPT’s responses. In general, participants found the responses acceptable; however, some noted awkward phrasing or grammatical inaccuracies, particularly in Turkish. This finding suggests that while ChatGPT’s Turkish quality is acceptable, it may struggle with accuracy and fluidity. These limitations can diminish the overall quality of the language output, particularly in more complex or nuanced tasks. One participant mentioned: *“So*,* the suggestion it gave always felt like something I’d gotten from a website. It didn’t make me feel like I was talking to a friend or a family member*.”

**Fig. 3:**
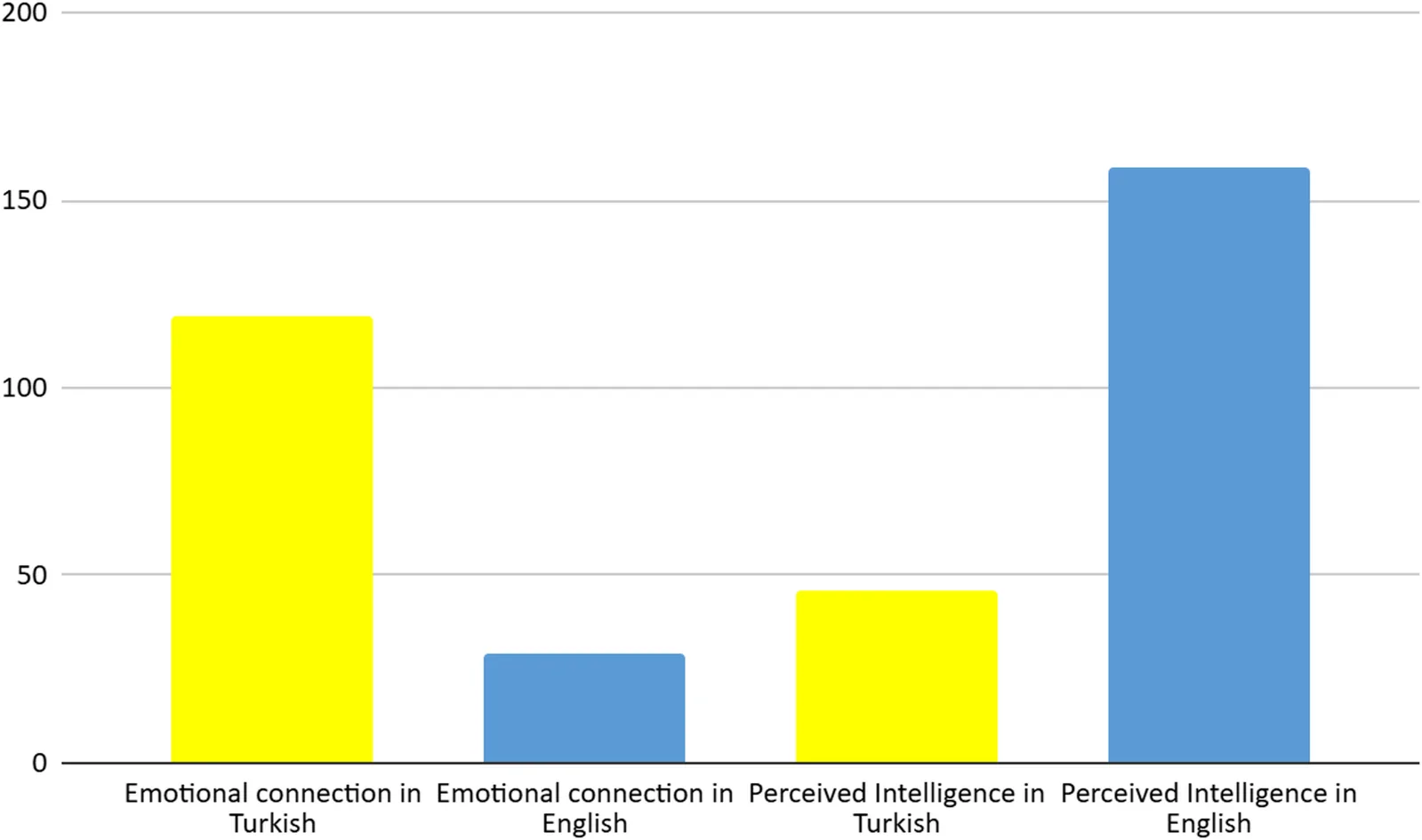
Comparison between languages

This finding suggests that while participants generally considered ChatGPT’s Turkish responses acceptable, they also perceived limitations in linguistic accuracy and conversational fluidity. This perception indicates that linguistic quality may play an important role in shaping users’ sense of social presence in human–AI interaction. This theme explores the strengths and limitations of ChatGPT’s linguistic capabilities in Turkish and shows a negative correlation with participants’ age (*r = −* .43, *p* = .012). Taken together, findings reveal an asymmetry between language use and perceived functionality. Participants reported a stronger emotional connection when interacting with ChatGPT in Turkish, their native language. However, perceived intelligence was markedly higher in English (See Fig. 3).

This duality suggests that emotional resonance is associated more with native language, while cognitive authority is associated with the foreign language, namely English. Thus, non-native English speakers may face a structural disadvantage. They use their native language to build emotional trust; however, they perceive ChatGPT as more competent in English for tasks requiring expertise. In contrast, native speakers of English might benefit more from both emotional closeness and intellectual efficiency while using their mother tongue [43].

## Discussion

MET [3] suggests that humans will likely treat computers and media as humanlike social agents. This study aimed to explore whether this was the case for AI systems and whether participants would respond to AI in a similar way to how they would interact with actual people. The theory also implies that users apply the same social and psychological rules to human-to-human and human-computer interactions. Results support these ideas, as many participants attributed humanlike qualities to ChatGPT and even asked it explicitly to ‘talk like a psychologist’ or ‘friend.’

Moreover, the findings showed a negative correlation between English proficiency and human-likeness. Thus, participants with higher English proficiency were less likely to perceive ChatGPT as humanlike in Turkish. This is an important finding that can be used to explore the social agent role of AI. When interacting in English, participants projected humanlike qualities onto the AI and likely attempted to compensate for the cognitive effort required when using their foreign language. Also, the positive correlation between linguistic fluency and emotional connection in English may imply that users with a stronger command of English experience a deeper connection with ChatGPT, driven by richer vocabulary and subtler emotional expression.

Also, ChatGPT’s linguistic capabilities in Turkish show a negative correlation with participants’ age. One possible explanation is that younger users are more accustomed to interacting with AI-based systems and may therefore be more tolerant of minor linguistic inaccuracies. In contrast, older participants may place greater emphasis on grammatical correctness in Turkish, making them more attentive to perceived errors in AI-generated responses.

Uncanny Valley was positively correlated with Emotional Attachment in English. This finding might suggest that participants who feel more emotionally attached during English interactions tend to experience a more pronounced Uncanny Valley effect. This can also be interpreted as a result of cognitive load, since participants interacted in a non-native language, which might have increased their emotional sensitivity and led to a higher perception of uncanny features.

Cultural fluency is defined as the capacity to cross cultural boundaries and communicate effectively, close to a native speaker’s understanding [44]. It is necessary for AI systems to be culturally aware to enhance user experience [45]. It is also important to note that cultural identity shapes how people incorporate AI into constructing the self in relation to others. According to research, people from individualist cultures may view AI as external to the self and are more likely to perceive AI as a threat to their uniqueness, autonomy, and privacy. In contrast, people from collectivistic cultures may perceive AI as an extension of the self, leading to a more positive perception of AI systems [46]. Therefore, AI’s effectiveness also depends on an understanding of cultural nuances. We also performed a correlational analysis for this theme. We found that it was negatively correlated with the theme of general emotional connection (*r* = − .41, *p* = .019). This finding might suggest that users who cared more about norm violations or cultural misalignments were less likely to form emotional bonds with ChatGPT. Thus, general emotional connection was correlated with cultural recognition in human–AI interactions.

Moreover, reported emotional attachment in Turkish was stronger, and participants felt warmer and more humanlike when interacting in their native language. Communicating in one’s native language is likely to elicit deeper emotional resonance, greater authenticity, and stronger self-expression. This finding aligns well with the MET, as the theory suggests that cultural and emotional appropriateness are important for being perceived as humanlike [3]. This may explain why participants reported greater general emotional connection and perceived ChatGPT as more humanlike when interacting in Turkish. Also, Keysar, Hayakawa, and An [47] argue that thinking in a foreign language may reduce decision biases, as it creates greater emotional and cognitive distance than the first language. Foreign-language communication often involves more conscious processing and less emotional spontaneity, which may lead to a more functional or transactional perception of the interaction.

Interacting in Turkish evoked a greater emotional connection, possibly due to cultural alignment, familiarity, and the ways language shapes users’ social perceptions of ChatGPT. Participants reported that interactions in Turkish generated stronger emotional resonance, warmth, and a sense of interpersonal familiarity. In contrast, interactions in English were often perceived as more efficient, competent, and intelligent. Rather than representing a contradiction, this pattern points to a structural tension in bilingual AI interactions.

Specifically, non-native English speakers appear to experience a trade-off: the language that fosters a stronger emotional connection (Turkish) is also the language in which the AI system is perceived as less capable, whereas the language associated with greater perceived competence and intelligence (English) tends to feel more affectively distant. The stronger general emotional connection in native-language interactions might also be related to the key concepts from positive psychology.

For example, emotional resonance and the intensity of emotional connection are likely to be higher when individuals use their first language. Pavlenko and colleagues argue that this phenomenon may be linked to early emotional development and autobiographical memory [48]. Because participants felt more emotionally comfortable interacting in their native language, they were more likely to perceive AI as humanlike.

On the other hand, interactions in English resulted in a more robotic perception of AI. Gabbiadini et al. [49] suggested that emotions about human-AI interactions are based on how users perceive AI. If the user perceives AI as a robot or an out-group, negative emotions are more likely to arise. Therefore, people use avoidance mechanisms to deal with these emotions, thereby increasing threat perception. Therefore, AI tools should leverage language skills to build rapport and avoid cultural mistrust.

Studies show that gender, race, and even the tone of voice affect trust in AI systems [50, 51]. Still, NLP (Natural Language Processing) and AI systems are often trained on data that represents WEIRD (Western, Educated, Industrialized, Rich, Democratic) populations [52]. Hovy and Spruit [52] suggested that this underrepresentation and exclusion might result in a technological divide in which minorities and non-English speakers have more difficulty accessing AI technologies. This idea aligns with the results of the current study. Therefore, Explainable Artificial Intelligence (XAI) systems should be encouraged so that AI tools can explain their behavior within a social context and enable users to understand how the model works [53]. Barredo Arrieta et al. [54] argue that transparency and explainability are not simple techniques; rather, they are ethical obligations for developers to overcome linguistic racism.

Farina and Lavazza [28] argue that LLMs risk amplifying the hegemony of English, potentially marginalizing minority languages and cultures. They evaluated the situation with current large language models (LLMs), as they pose risks of amplifying English hegemony. Authors suggest that these tools can potentially homogenize communication and overshadow linguistic diversity since LLMs are often trained on English-language data.

Some studies in the literature have collected data from both English-speaking and non-native English-speaking participants. For example, a study on Google Home found that both user groups were satisfied with the device [43]. However, native English speakers had a better user experience, and preliminary findings also showed patterns about cultural distinctions in English expression and system engagement. Similarly, Helin and colleagues [55] advocated that voice assistants transition from monolingual by default to multilingual by default. Authors [28] argue that these tools may marginalize minority languages and cultures and suggest that governments and international bodies should focus on language pluralism and protect minority cultures in the digital sphere. Taken together, these findings highlight the role of language not merely as a medium but as a modifier of user experience. Language can influence both how AI is perceived and how users relate to it.

### Limitations and future research

There are several limitations to this study. With 33 participants, the study exceeds the sample size conventionally associated with IPA, which typically involves 6–10 cases. This decision reflects the dual requirements of the study’s hybrid design, which incorporates both qualitative (IPA) and quantitative (correlational) components. Future studies adopting a purely IPA design may benefit from working with smaller, more homogeneous samples to enable richer individual-level analysis.

In addition, the participants in this study were bilingual adults who were native Turkish speakers with self-reported English proficiency at the upper-intermediate level or higher. This may limit the generalizability of the findings to speakers of other language pairs. Future studies would also benefit from incorporating standardized proficiency assessments to allow for more precise sample characterization. Furthermore, there were no native English speakers in the current sample, which may have influenced participants’ perceptions of AI-generated responses in English. Future research should compare these findings with native English speakers to disentangle language proficiency effects from AI performance differences, since some of the observed effects can also occur in non-AI communication contexts [56].

A further limitation concerns the design of the interaction prompts. Although both prompts were situated within the same broad thematic domain of psychological coping and well-being, they were not lexically identical across the two languages. This decision was deliberate: presenting participants with the same prompt twice would have introduced boredom and fatigue effects, social desirability bias, and demand characteristics, thereby compromising the naturalistic quality of the data. Future studies may wish to explore counterbalanced or matched prompt designs. Also, the order of prompts given before the interviews could have affected the results.

Future studies should also be conducted with different linguistic backgrounds and cultural contexts. Additionally, the only AI system used in this study was ChatGPT, and results may not fully capture the diversity of AI interactions. Conducting the study with a broader range of AI systems and tasks to assess the same variables would also be interesting.

## Conclusion

This study investigated how bilingual users perceive ChatGPT based on their language preferences, revealing a theoretically significant divergence between two dimensions of human–AI perception: emotional connection and perceived intelligence. While participants attributed greater intelligence and efficiency to English interactions, they reported stronger emotional resonance, warmth, and anthropomorphic engagement in Turkish. This bifurcation constitutes a theoretical extension of MET into multilingual contexts, demonstrating that the social and emotional responses users attribute to AI systems are not uniform but are systematically moderated by language and cultural context.

This finding advances the existing literature in several important ways. First, it challenges the implicit assumption in much of the MET-based literature that social responses to technology are relatively stable across users and contexts. The present study shows that language fundamentally shapes the nature and quality of the human–AI relationship.

Second, it identifies a structural trade-off faced by non-native English speakers: the language that affords greater emotional connection is also the language in which AI systems perform less capably, while the language that affords greater perceived intelligence is experienced as affectively distant. This trade-off has implications not only for user experience but also for equity of access. Non-native speakers are systematically disadvantaged in their ability to benefit from both the emotional and cognitive affordances of AI systems simultaneously. Third, the study offers a rare, phenomenologically grounded qualitative account of multilingual AI interaction, complementing the predominantly quantitative literature on cross-linguistic AI performance with rich, experience-near data on how these disparities are lived and felt by users.

The practical implications of this study are crucial for developers aiming to improve AI systems’ cultural adaptability. Linguistic proficiency and cultural alignment should be central considerations in both the design and evaluation of AI systems. AI tools should tailor conversational style, tone, and content based on users’ native language and respond in ways that align with users’ emotional and social expectations. For example, ChatGPT could use more friendly, culturally familiar expressions in Turkish, since Turkish culture values warmth and sincerity. Enhancing AI’s cultural fluency and emotional responsiveness in multiple languages could lead to more inclusive and equitable systems, ensuring that non-native English speakers can access both the emotional and intellectual affordances of AI that native English speakers currently take for granted.

In conclusion, this study demonstrates that language is not merely a medium of communication in human–AI interaction. It is a fundamental determinant of how users perceive, relate to, and derive value from AI systems. Multilingual AI systems must go beyond mere translation to offer emotionally and culturally nuanced outputs that reflect the full diversity of their users’ linguistic and cultural identities. The findings advocate for linguistically adaptable AI design that takes local expressions, idioms, and emotional norms into account, and call for greater investment in the development of high-resource AI capabilities across a broader range of languages.

## Data Availability

Data is available upon reasonable request.

## Footnotes

1
_All participant quotes were originally produced in Turkish and translated into English by the author. Parenthetical annotations marked with the author’s initials (MAB) indicate translator’s notes inserted to preserve the cultural or linguistic meaning of terms that do not have a direct English equivalent. These annotations do not alter the substance of participants’ accounts_.
